# The use of induced pluripotent stem cells as a platform for the study of depression

**DOI:** 10.3389/fpsyt.2024.1470642

**Published:** 2024-10-09

**Authors:** Javier Villafranco, Gabriela Martínez-Ramírez, Roxana Magaña-Maldonado, Anna Paola González-Ruvalcaba, Adolfo López-Ornelas, Iván Velasco, Enrique Becerril-Villanueva, Lenin Pavón, Enrique Estudillo, Gilberto Pérez-Sánchez

**Affiliations:** ^1^ Laboratorio de Reprogramación Celular, Instituto Nacional de Neurología y Neurocirugía Manuel Velasco Suárez, Ciudad de México, Mexico; ^2^ Laboratorio de Psicoinmunología, Instituto Nacional de Psiquiatría Ramón de la Fuente Muñíz, Ciudad de México, Mexico; ^3^ Facultad de Estudios Superiores Iztacala, Universidad Nacional Autónoma de México (UNAM), Tlalnepantla, Mexico; ^4^ División de Investigación, Hospital Juárez de México, Mexico City, Mexico; ^5^ Hospital Nacional Homeopático, Hospitales Federales de Referencia, Mexico City, Mexico; ^6^ Instituto de Fisiología Celular - Neurociencias, Universidad Nacional Autónoma de México, México City, Mexico

**Keywords:** iPSCs, stem cells, depression, neurons, glia, astrocytes

## Abstract

The neurobiological mechanisms underlying major depressive disorder (MDD) remain largely unexplored due to the limited availability of study models in humans. Induced pluripotent stem cells (iPSCs) have overcome multiple limitations of retrospective clinical studies, contributing to a more detailed understanding of the molecular pathways that presumably contribute to the manifestation of depression. Despite the significant progress made by these study models, there are still more formidable challenges that will eventually be addressed by these platforms, as further studies may eventually emerge. This review will examine the most recent advances in the comprehension of depression by using human neurons and non-neuronal cells derived from induced pluripotent stem cells of patients with depression. This study highlights the importance of using these platforms to increase our knowledge of depression and address this psychiatric disorder more efficiently.

## 1 Introduction

Major depressive disorder (MDD) is the main psychiatric condition affecting people worldwide. Despite the large knowledge that allows its diagnosis, there are remaining issues that hamper their efficient treatment in many patients. The inefficient treatment of depression discloses a lack of knowledge regarding the cellular and molecular mechanisms that underlie this condition. Although animal models have contributed to a better comprehension of this disease, the use of human cells as platforms to study cellular and molecular aspects of depression represents a valuable tool to identify key aspects of their functioning on depression. Regarding this issue, human induced pluripotent stem cells (iPSCs) are a valuable platform to model depression, understand the way neurons and other cells behave in this condition, and test several promising therapeutic agents on them (1). This novel research field represents a promising way through which many underlying mechanisms of depression could be unraveled. The aim of this mini-review is to analyze the most recent and pioneering works exploring the functioning of neurons and other overseen non-neuronal cells such as oligodendrocytes, astrocytes, neuronal precursors, and fibroblasts, derived from iPSCs of patients with this disorder.

## 2 General aspects of depression

Major depressive disorder (MDD) is defined as the persistence of a depressed mood, loss of interest or pleasure in previously pleasurable activities (anhedonia), recurrent thoughts of death, and the onset of physical and cognitive symptoms (1). Since there is not a single symptom that is pathognomonic of depression by itself, its definition refers to a disorder integrated as a syndrome that causes functional impairment; some symptoms are anhedonia, reduced sex drive, diurnal variation of symptoms, guilt, fatigue, loss or gain of appetite or weight, and insomnia. For a diagnosis of depression, symptoms must be present throughout the day, last for two weeks or more, and not be explained by another medical condition (2). Depression not only causes a deterioration in mental health but also has been associated with an increased risk of developing other conditions, which, in addition, have higher mortality rates than the general population, reaching up to 60–80% (3, 4).

### 2.1 Epidemiology

The Global Burden of Diseases, Injuries, and Risk Factors Study (GBD) estimates a prevalence of 970.1 million cases in 2019, corresponding to an increase of 48.1% between 1990 and 2019. This psychiatric condition is more common in women than in men and in regions such as North America, South America, Australia, and Asia. Moreover, depression increases the risk of suicide, which was the 18th leading cause of death in the GBD 2019 (5). Therefore, depression is on the rise and has become a significant burden for healthcare systems. The emergence caused by the COVID-19 pandemic has contributed to increased conditions affecting people’s mental health. As a result, the prevalence of depression has increased, with women and young people being the most affected. After the pandemic, the prevalence increased to 3152 cases per 100 000 people, affecting 246 million people. This represents 53.2 million new cases during the pandemic (6). Older adults are a vulnerable population for the development of depression, with one meta-analysis estimating a prevalence of 35.1%. In addition, older people in low- and middle-income countries have a greater prevalence of depression than those in high-income countries, which may be explained by low levels of education, inadequate pensions, limited access to health services, and poor economic conditions (7).

## 3 The problem: lack of knowledge with a prospective approach in the human context

Animal models have long been utilized as a fundamental component of psychiatric research, with the objective of replicating the intricate nature of these diseases. However, the complexity of the human nervous system and behavior differ significantly from those of other mammals, and the polygenic nature of psychiatric diseases poses significant limitations to the efficacy of these models (8). Research on psychiatric diseases is hampered by the inability to obtain human brain biopsies or culture primary neurons from humans. In addition, the study of postmortem brains presents some difficulties and does not provide precise information about the onset of the disease (9). Given the ongoing challenges in modeling human diseases via current laboratory assays or animal models, obtaining patient-specific iPSCs from their somatic cells has emerged as a promising alternative. This approach could provide a more profound understanding of the pathogenesis of psychiatric diseases through disease modeling and the design of more efficacious therapeutic interventions. Furthermore, it could also facilitate the acceleration of drug discovery and improve the efficacy of drug testing (10–12). The practical implications of this technology are opening new possibilities in personalized medicine, revolutionizing the field of medicine and drug development.

## 4 Use of iPSCs as a platform for studying depression

iPSCs represent a valuable tool for advancing our understanding of neuropsychiatric diseases. Initially, reprogramming human somatic cells into iPSCs was achieved through the integration of retroviral vectors, which delivered the reprogramming genes Oct3/4, Sox2, Klf4, and c-Myc (10, 13). These methodologies have permitted cellular reprogramming to yield neurons derived from different types of patients including those with depression, thereby providing a valuable instrument for the assessment of the development and progression of this psychiatric disorder (14).

## 5 Neurons derived from MDD patients

Neurons derived from the iPSCs of MDD patients have provided valuable information regarding the underlying mechanisms that promote depression. Recent studies have demonstrated that neurons derived from MDD patients display alterations in their electrophysiological properties in addition to impairments in their bioenergetic and mitochondrial functions (15, 16). Triebelhorn et al. demonstrated that neurons derived from the fibroblasts of MDD patients exhibited a smaller cell size than those derived from healthy subjects. Additionally, these neurons demonstrated lower membrane capacitance, resting membrane potential, and sodium current. Moreover, one of the main characteristics of these neurons is that they exhibit spontaneous activity. These electrophysiological alterations may be attributed to mitochondrial dysfunction in neuronal precursors resulting from a diminished function of the mitochondrial oxidative phosphorylation system (15).

Notably, forebrain neurons derived from MDD patients that do not respond to antidepressant treatments exhibit greater excitability and responsiveness to serotonin (5-HT) than do those derived from responsive patients and healthy controls. This is evidenced by an increase in calcium signaling following 5-HT stimulation. This information is consistent with the observation that neurons derived from nonresponsive MDD patients have increased protein levels of the HTR_2A_ and HTR_7_ receptors. Furthermore, blockade of these receptors has been shown to inhibit neuronal hyperexcitability (17). It remains to be determined whether these alterations are homeostatic processes that counteract the cellular and molecular mechanisms that promote depression or whether impairments in forebrain neurons contribute to the development of this psychiatric disorder (18). Notably, neurons derived from MDD patients with impaired mitochondria also demonstrate heightened activity (15), indicating that the elevated neuronal activity observed in nonresponsive patients may be partially attributable to mitochondrial damage.

Further studies focused on serotonergic neurons demonstrated that this neuronal type derived from nonresponder MDD patients displays longer neurites, which suggests altered plastic processes that could contribute to the symptomatology of depression (19). In accordance with this evidence, another research group reported that cortical neurons derived from nonresponsive MDD patients presented a reduction in synaptic connectivity and an impairment in the number and types of dendritic spines. Additionally, these cortical neurons displayed novel differentially expressed genes, including NPPB, LRRC15, MICB, CHMP4C, and ITGA2, which suggests their potential use as biomarkers for MDD patients who are nonresponsive to antidepressants (20).

GABAergic neurons derived from MDD patients also display plasticity impairments, as evidenced by their hyperexcitability and impairment of calcium signaling. Additionally, these neurons also exhibit a distinct morphology compared with those derived from healthy controls, with a greater number of neurite ramifications. This suggests alterations in structural plasticity. Further experiments confirmed the downregulation of HTR_2_ in these neurons. Pharmacological treatment with the HTR_2_ agonist Trazodone was found to restore the altered activity and neurite branching of GABAergic neurons (18).

**Figure 1:**
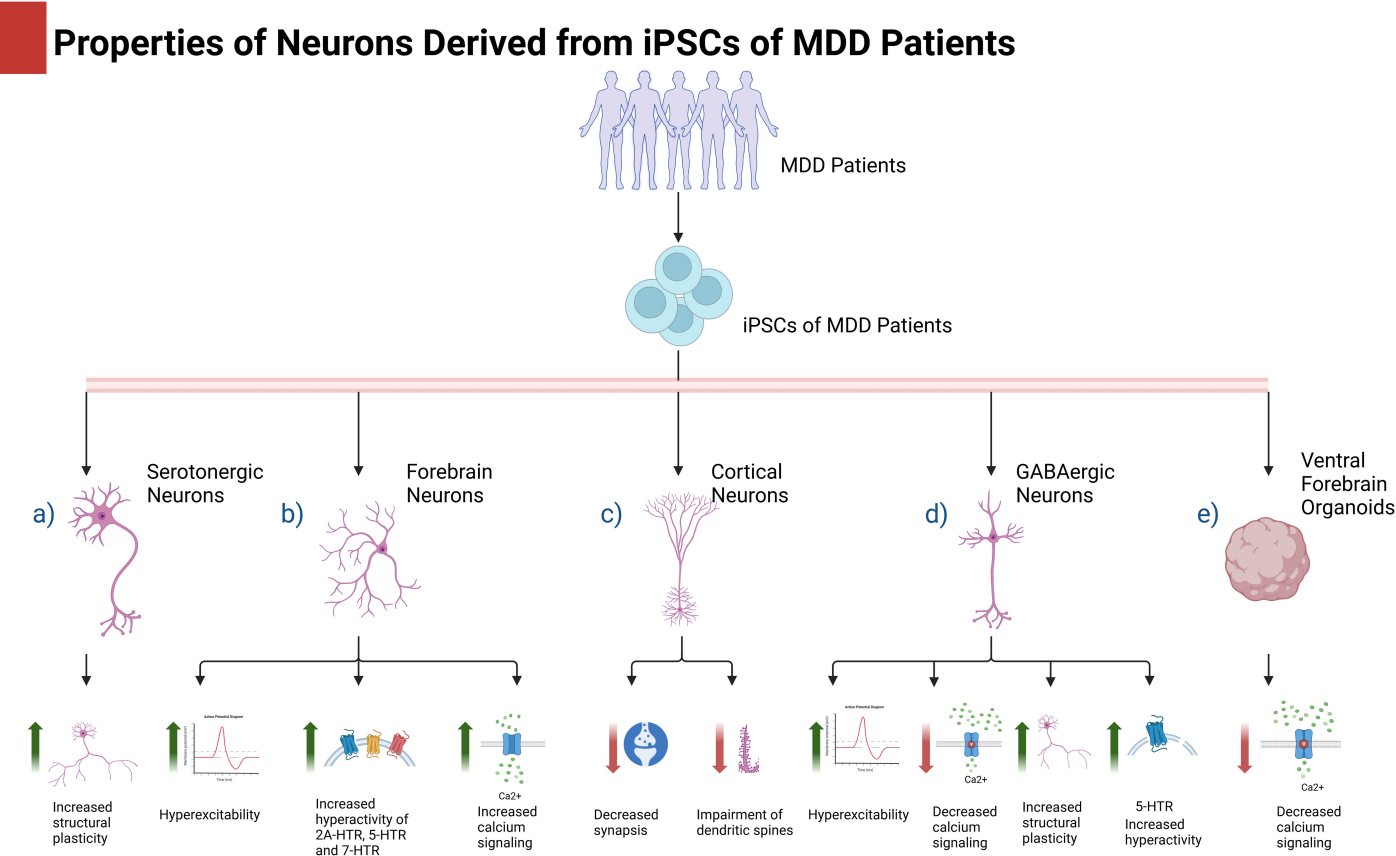
iPSCs from depressive patients and their use in depression. iPSCs from depressive patients can differentiate into different types of neurons or brain organoids where several molecular and cellular aspects can be studied. Evidence indicates that depression affects the biological properties of neurons in different manners depending on their phenotype. **Figure 1** created with BioRender.com.

Collectively, these reports demonstrate that distinct morphological patterns, electrophysiological properties, and differential gene and protein expression can be observed in various types of neurons derived from patients diagnosed with MDD (**Table 1**; **Figure 1**). These findings highlight the significant utility of cell reprogramming and the use of iPSCs in elucidating the pathophysiology underlying MDD. Although relevant, the results from two-dimensional cultures require complementation with other study models to understand complex psychiatric conditions such as depression and obtain robust results that could allow more accurate interpretations of the mechanisms underlying the development of depression and resistance to antidepressive treatments.

**Table 1:** Two-dimensional and three-dimensional cultures as a model for the study of MDD.

Induced cell type	Study Platform model: 2D-*in vitro* or 3D-spheroids	Clinical and pharmacological conditions	Biological effects	References
neural progenitor cells (NPCs), astrocytes and neurons	2D	Two specific patients: -antidepressant non-responding MDD patient (Non-R) -mitochondriopathy patient (Mito)	Neurons derived from MDD patients showed a smaller cell size than those derived from healthy subjects. The biophysical properties and function of Non-R and Mito patients’ neurons are different from their corresponding controls. Mito patients and a broader MDD cohort had decreased respiration and mitochondrial function, while Non-R patients had increased respiratory rates, mitochondrial calcium, and resting membrane potential	(15)
NPCs and iPSC-derived neurons	2D	Patients received antidepressant medication and were nearly in remission	MDD patient-derived iPSC-neurons had lower membrane capacitance, reduced hyperpolarized membrane potential, increased Na+ current density and spontaneous electrical activity	(16)
Forebrain neurons	2D	Cases of Selective serotonin reuptake inhibitors (SSRI)-responsive (remitters, R) and SSRI-resistant (nonremitters, NR) patients	NR patient-derived neurons showed serotonin-induced hyperactivity downstream of upregulated excitatory serotonergic receptors, in contrast with healthy subjects and R patient-derived neurons. Altered serotonergic neurotransmission is linked to SSRI resistance	(17)
GABAergic interneurons (GINs) and ventral forebrain organoid	2D 3D	Major depressive disorder with suicide behavior (sMDD)	Serotonergic receptor subtype 2C (5-HT2C) is decreased in sMDD GIN, contributing to altered neuronal activity. Besides, these GINs showed increased neurite arborization, neural firing, and reduced calcium signaling	(18)
Cortical neurons	2D	Bupropion effect on remission or non-remission in Sequenced Treatment Alternatives to Relieve Depression (STAR*D) patients without citalopram response	Responder-derived neurons had longer spines, different spine length distribution, and increased specific spine-type length. These neurons showed differential gene expression patterns, and the identified genes could be used as predictive biomarkers in bupropion response	(20)
BrainSpheres (Astrocytes, neurons, and oligodendrocytes)	3D	Developmental neurotoxicity (DNT) model in organoids - paroxetine exposure.	Paroxetine induces alterations during neurodevelopment. These alterations include a reduction in the expression of synaptic markers, neurite outgrowth and oligodendrocyte cell population. BrainSpheres constitute a novel tool to analyze compounds with potential developmental neurotoxicity	(21)
Dopaminergic (DA) neurons	2D	Ketamine effect on human DA neurons differentiated from human iPSCs	Ketamine dose-dependently promotes structural plasticity, showing increased soma size and enhanced dendritic outgrowth. These effects are mediated through the activation of alfa-amino-3-hydroxy-5-methyl-4-isoxazolepropionic acid (AMPA) receptors, which induces mammalian target of rapamycin (mTOR) signaling driven by brain-derived neurotrophic factor (BDNF)	(22)
Heterogeneous mixed populations of neurons, astrocytes, and oligodendrocytes	2D	Arylsulfatase A (ARSA) enzymatic deficiency in metachromatic leukodystrophy (MLD) patients	MDL patients showed altered composition and accumulation of sulfatide during differentiation to glial and neuronal cells. These changes were associated with apoptosis, oxidative stress, and expansion of the lysosomal compartment. ARSA deficiency promoted an atypical astroglial and neuronal differentiation of human MLD iPSCs-NPCs, showing a reduced level of astroglial and oligodendroglial markers, a reduced number of neurons, and a disorganized neuronal network	(23)
Astrocytes	2D	Patients with depression scored via HAMD-17 and QIDS-C16 rating scales. Model of chronic stress- exposure to cortisol	Chronic cortisol exposure in astrocytes derived from MDD patient iPSCs induced differential gene expression related to GPCR binding, ion transport regulation, and chemical synaptic transmission. This chronic exposure might affect astrocyte morphology, cell adhesion, and extracellular matrix organization	(24)

To address this issue, three-dimensional cultures of cells from the nervous system, such as brain organoids, have the advantage of displaying spatial organization. Brain organoids self-organize into cell clusters similar to multiple structures of the human brain, which allows the study of the complex interactions between multiple cell types, such as neurons and glial cells, in multiple neurological and psychiatric diseases (8, 21–25).

A study using iPSCs from genetically isolated families with a high prevalence of mood and psychotic disorders, including MDD, successfully generated neural cells and 3D brain organoids (26). These organoids revealed significant rare genetic variants, particularly enriched in this population. Key findings included mutations in genes like CACNA1C (calcium signaling), SLC6A4 (serotonin transport), and BDNF (synaptic plasticity). For example, organoids with CACNA1C mutations showed disrupted calcium signaling, essential for neurotransmitter release, while SLC6A4 alterations were linked to abnormal serotonin signaling, a critical pathway in depression.

These findings suggest that iPSC-derived organoids could be pivotal in uncovering the molecular mechanisms underlying treatment-resistant depression. Disruptions in signaling pathways, including mTOR and ERK, and altered serotonin and dopamine signaling indicate that these genetic variants significantly affect neuronal communication. This understanding could lead to more personalized treatment strategies, with therapies designed to target specific molecular dysfunctions identified in a patient’s genetic profile. By focusing on these pathways, new therapeutic approaches for MDD, especially for those resistant to conventional treatments, could be developed, advancing towards more precise and individualized mental health care (26).

Notably, organoids of the ventral forebrain derived from the iPSCs of MDD patients also contain GABAergic neurons. Remarkably, in this 3D model, neurons display impaired calcium signaling as in 2D cultures, thus demonstrating consistent results between both types of cell culture. These results highlight the importance of 3D cultures as a plausible experimental approach that, when combined with 2D cultures, can provide robust results to strengthen and encourage the use of iPSCs as a study model for MDD (18). However, as the development of brain organoid research accelerates, it introduces significant ethical concerns, such as the possibility that these organoids could develop some level of consciousness or sentient-like qualities, and the variability and unpredictability inherent in brain organoid research, which compromises the quality and integrity of results derived from 3D cultures. These drawbacks underscore the pressing need for comprehensive ethical guidelines, which are essential to ensure that scientific advancements in this field are matched by thorough ethical considerations to protect human dignity and rights, and highly reproducible protocols for brain organoid production and application (27–30).

iPSCs are also suitable platforms for obtaining specific neuronal types and evaluating their effects at the cellular level or the efficacy of potential pharmacological treatments for depression. The use of iPSCs has allowed researchers to analyze the antidepressant effects of ketamine on dopaminergic neurons. Interestingly, ketamine induced structural plasticity in dopaminergic neurons, as their dendrite length, number, and soma area increased in response to ketamine. Further pharmacological experiments on these neurons demonstrated that this effect was mediated mainly by mTORC1 and was dependent on the activation of the receptors AMPA-R, D3R, and TrkB (22). These results provide novel insights into the poorly understood mechanisms that underlie the antidepressive effects of ketamine on dopaminergic neurons.

On the other hand, iPSCs also allow the study of neurodevelopmental processes by generating brain organoids that recapitulate multiple stages of neurodevelopment (31). These properties make it possible to analyze the potential adverse effects of the use of antidepressants during pregnancy, as studied by Zhong and colleagues, who reported that paroxetine alters the expression of synaptic markers and neurite outgrowth in brain organoids, thus suggesting an impairment of structural plasticity in neurodevelopmental stages (21).

The complex interaction between neuronal and nonneuronal cells makes a challenging task the identification and comprehension of the underlying mechanisms that cause depression. Therefore, investigating the role of human nonneuronal cells in depression is mandatory to design more effective strategies for its treatment.

## 6 Glial cells

Glial cells play important roles in the proper functioning of the central nervous system (CNS). They include oligodendrocytes, astrocytes, microglia, ependymal cells, Schwann cells and radial cells (32). These cells participate in the maintenance of brain homeostasis, providing support for neurons, and in several immunological processes in both the CNS and the peripheral nervous system (32). The proper functioning of the CNS requires a complex network of interactions between glial cells and neurons. Glial cells communicate through different pathways, such as intracellular calcium signals and the diffusion of chemical messengers (33), and they participate in various repair processes in the CNS (34). Glial cells are important for the metabolic relationship between glia and neurons, as they provide energy substrates to neurons (35) and for the maintenance of the blood–brain barrier, which regulates the chemical composition necessary for neuronal circuit function, synaptic transmission, and neurogenesis in the adult brain (36).

### 6.1 iPSC-derived glial cells

iPSCs are an interesting tool for the generation of glial cells for various applications in regenerative medicine and disease modeling. However, while iPSC-derived neuronal cells have been extensively studied, the use of iPSC-derived glial cells has been relatively limited to date (37). iPSCs have been successfully used to generate motor neurons (MNs) and glial cells from patients with amyotrophic lateral sclerosis (ALS), revealing the potential of iPSCs to model disease phenotypes (38). Moreover, iPSCs have been used to generate glial cells from various cell sources, such as periodontal ligament cells, highlighting their versatility in terms of differentiation (39). Cocultures of patient iPSC-derived neurons with iPSC-derived glial cells, such as astrocytes, have been proposed as a strategy to investigate neurodegenerative diseases such as frontotemporal dementia (40). In addition, glial progenitors and iPSC-derived oligodendrocytes have been used in studies to model diseases caused by demyelination due to genetic factors, highlighting the crucial role of glia in neurological disorders (23). Furthermore, the incorporation of glial cells into neuronal cultures derived from iPSCs has been suggested to reveal distinct phenotypes and novel research avenues in diseases such as sporadic Alzheimer’s disease (sAD) (41). Compared with pure neuronal cultures, cocultures of iPSC-derived neurons with glial cells have been shown to increase the development of electrophysiological parameters, indicating the importance of glial–neuronal interactions (42). Thus, iPSC-derived glial cells are promising tools for disease modeling studies and regenerative medicine and for obtaining a deeper understanding of the role of glia in psychiatric disorders.

### 6.2 iPSC-derived astrocytes from MDD patients

Astrocytes are associated with the pathophysiology of MDD; indeed, astrocyte dysfunction has been observed to be present in mental disorders, including suicide (43). The literature indicates that astrocytic dysfunction contributes to the impaired neuroplasticity observed in MDD (44). Astrocyte dysfunction also influences cognitive alterations in other diseases, such as schizophrenia and bipolar disorder (45). Astrocytes also modulate neurotransmission and neurovascular coupling (46); therefore, alterations in astrocyte function affect synaptic plasticity in the prefrontal cortex, a brain region associated with MDD (47). Furthermore, the involvement of astrocytes in the NLRP3 inflammasome has been identified as a potential contributor to the pathogenesis of MDD (48).

In 2021, Heard et al. (24) generated astrocytes from iPSCs derived from MDD patients. The objective of this study was to investigate the differential impact of stress caused by chronic cortisol exposure on astrocytes derived from the iPSCs of individuals with MDD compared with those derived from healthy subjects. Chronic exposure to cortisol, a stress hormone, is a model for studying stress-induced depressive behavior. The Quick Inventory of Depressive Symptomatology (QIDS) and the Hamilton Depression Rating Scale (HDRS) were used to identify and collect biopsies from individuals with severe MDD. The iPSC-derived astrocytes from patients and control subjects were treated with 5 µM cortisol every 48 h for 7 days. They identified a specific stress response transcriptome of astrocytes from MDD patient-derived iPSCs. These differentially expressed genes (DEGs) are associated with GPCR-binding ligands, synaptic signaling, ion homeostasis, and chronic responses to cortisol. The Heard et al. study offers a suitable model for furthering our understanding of the complex relationship between chronic stress and the development of MDD (24).

Recent evidence also indicates that astrocytes from patients with major depressive disorder (MDD), with or without mitochondrial impairments, display alterations in bioenergetics and morphology. Furthermore, alterations in respiration rates, oxygen consumption, calcium content, cell size, and ROS have been observed in these astrocytes (15). Notably, fibroblasts from MDD patients also exhibit alterations in processes related to mitochondrial function, suggesting that these cellular impairments are also present in cells that do not reside within the nervous system (15).

### 6.3 Impact of paroxetine on oligodendrocytes in a 3D human brain model

Although oligodendrocytes play a pivotal role in neuronal communication, the literature on their role in MDD is limited. A pioneering study utilizing an iPSC-derived 3D human brain model (BrainSpheres) demonstrated that the antidepressant paroxetine reduces the number of oligodendrocytes in brain spheres, indicating the potential adverse impact of this drug on oligodendrocyte production during human neurodevelopment (28). This type of study enables us to assess more thoroughly the differential effects of antidepressants on cells of the central nervous system. Further research employing induced iPSCs as a study model will provide valuable insights into the adverse effects of these drugs.

### 6.4 Neuronal precursor cells

In addition to differentiated cells, neural precursors derived from the iPSCs of MDD patients also display alterations related to this psychiatric condition in terms of mitochondrial function and bioenergetic profile, such as a decrease in oxygen consumption, proton leakage, cell surface area, and basal and maximal respiration. These changes are also dependent on whether patients exhibit genetic alterations in mitochondria (15, 16).

## 7 Concluding remarks

Although MDD is among the most prevalent psychiatric disorders, there is still a paucity of data regarding the molecular mechanisms underlying its onset and progression. Animal and retrospective studies have made substantial contributions to a better comprehension of this psychiatric condition. Nevertheless, there is a pressing need to develop novel platforms to elucidate the manner in which human neurons and nonneuronal cells behave in MDD, and to further comprehend its pathophysiology. The study of human neurons, glial cells, and other nonneuronal cells derived from the iPSCs of MDD patients has demonstrated their potential to elucidate the cellular and molecular aspects of MDD, which could lead to a detailed understanding of this disorder. Consequently, iPSCs utilization in future studies will increase the quantity and quality of information regarding depression and represents a promising avenue for improving the design of treatments and therapies for this disorder.
